# Supplementary material for: Activation of GPR56, a novel adhesion GPCR, is necessary for nuclear androgen receptor signaling in prostate cells
Source: PLoS One. 2020 Sep 3;15(9):e0226056. doi: 10.1371/journal.pone.0226056 (PMC7470385; doi:10.1371/journal.pone.0226056)
Supplement: S1 Table — (DOCX) [file pone.0226056.s001.docx]

**S1 Table. Clinical characteristics of 25 PCa patients**

| **Total no. of patients (N=25)** | | |
| --- | --- | --- |
| 1. | Age at diagnosis (years) | 61-72 |
| 2. | PSA (ng/ml)  < 10  10-20  >20 | 18.7-25.8  10  12  3 |
| 3. | Gleason score  2-6  7-10  Unknown | 14  8  2 |
| 4. | Tumor stage identification  T1-T2/No/Mo  T3-T4/No/Mo  unknown | 18  5  2 |
| 5. | Node positive disease  Present  Absent  Unknown | 7  15  3 |
| 6. | Distant Metastases  Present  Absent  Unknown | 4  19  2 |
