# Supplementary figures and images for: Activation of GPR56, a novel adhesion GPCR, is necessary for nuclear androgen receptor signaling in prostate cells
Source: PLoS One. 2020 Sep 3;15(9):e0226056. doi: 10.1371/journal.pone.0226056 (PMC7470385; doi:10.1371/journal.pone.0226056)

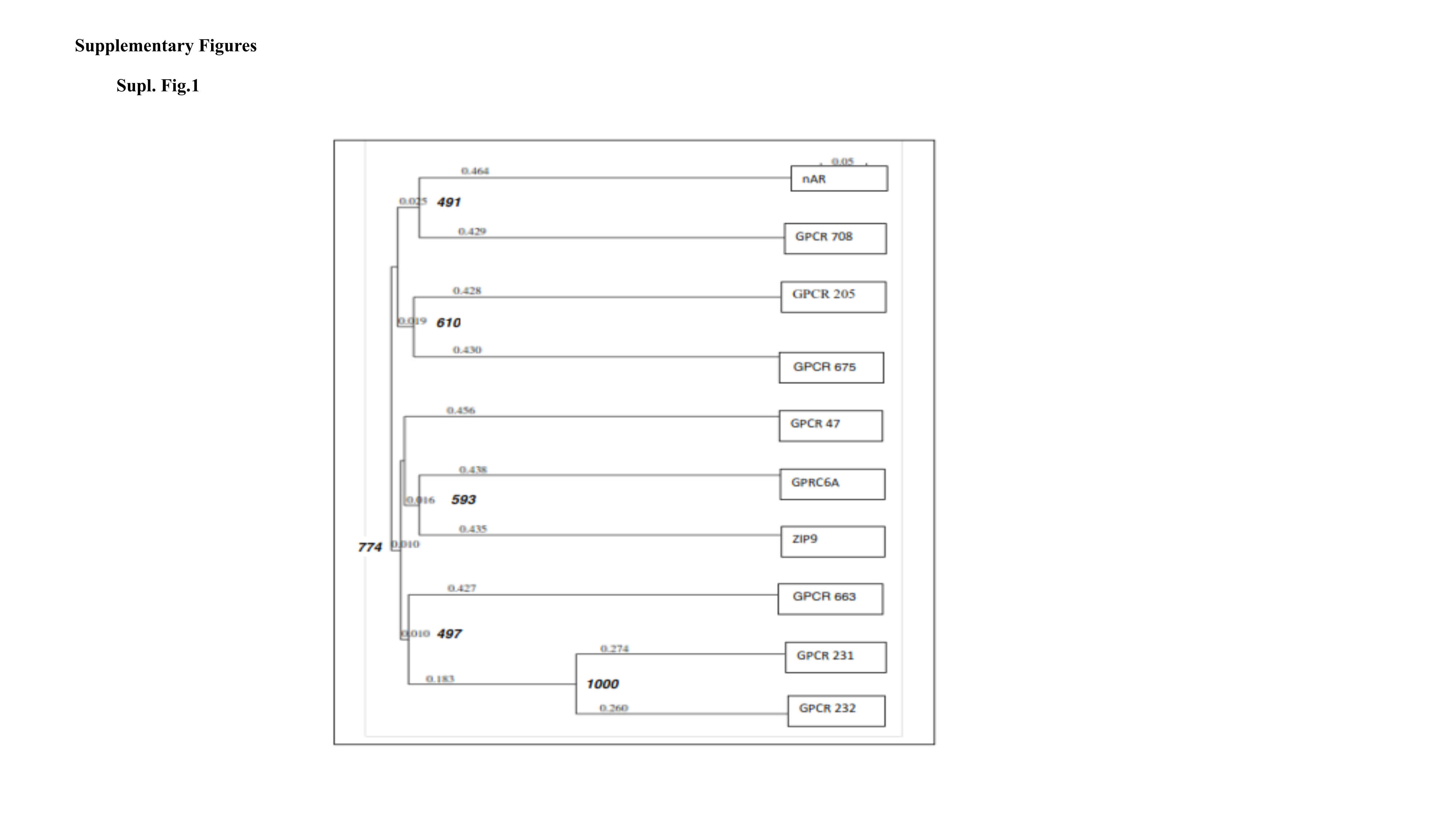

Supplement: S1 Fig — The numbers in italics are the bootstrap values. Putative membrane ARs (GPRC6A, ZIP9 and GPCR 663) proposed in other studies were also included to assess the relatedness among their sequences. (TIF) [file pone.0226056.s002.tif]

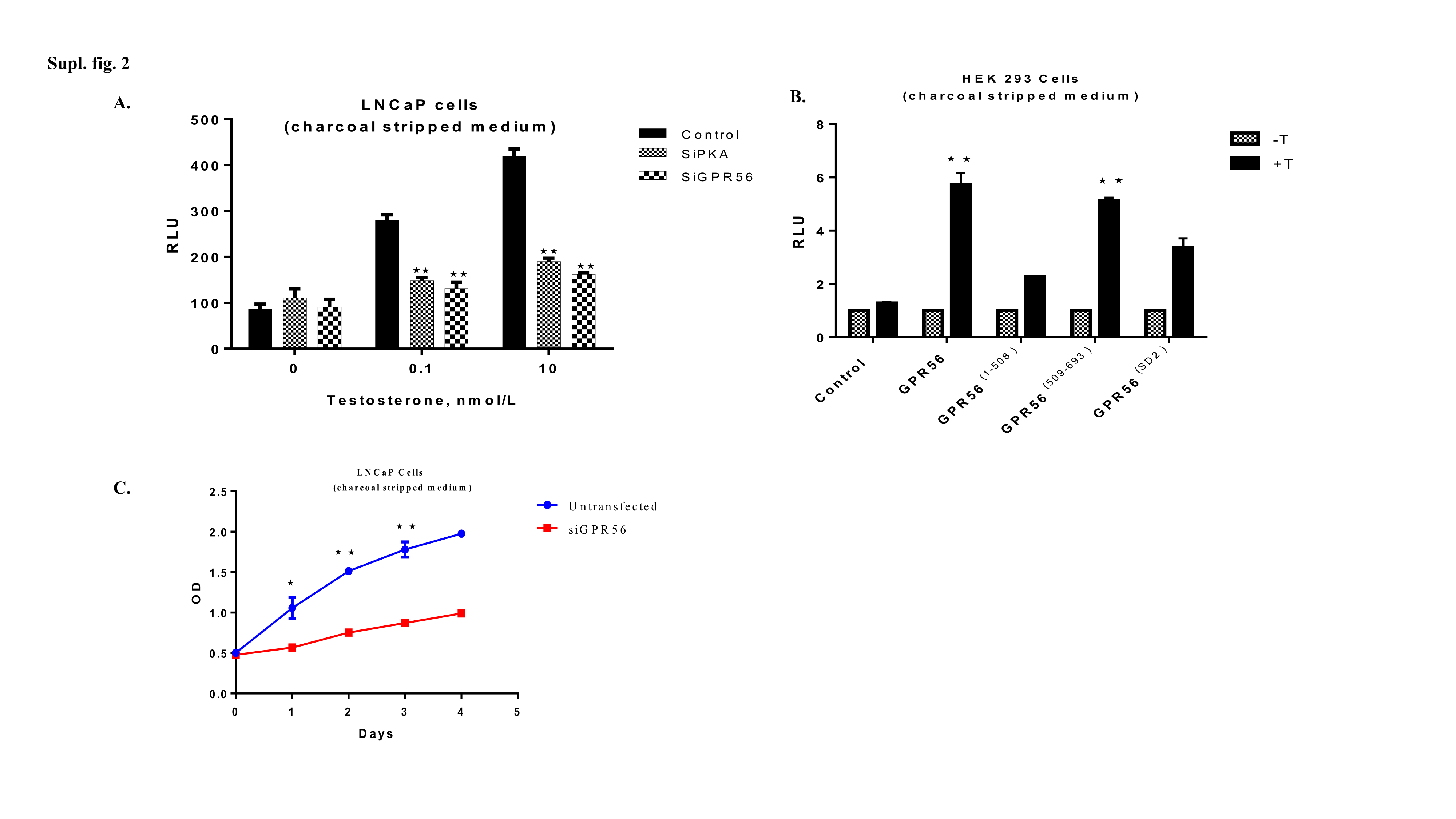

Supplement: S2 Fig — A) Inhibition of AR transcription by knocking down GPR56 and PKA expression using charcoal stripped medium. LNCaP cells transfected with ARE-Luc (1μg) reporter plasmid with scrambled siRNA(100nM) or siGPR56 (100nM) and siPKA (120nM). The cells were treated with 0.1 and 10nM testosterone 24 h after transfection period. Values are mean ±S.D. from three independent experiments. * p< 0.01, ** p<0.001, two-way anova test. B) AR transcription analysis of GPR56-NT, GPR56-CT, and GPR56 double mutant. HEK293 cells transfected with ARE-Luc (1μg) reporter plasmid with GPR56, GPR56 N terminus mutant, GPR56 C terminus mutant, GPR56 double mutant (SD2) plasmids using charcoal stripped medium. The cells were treated with 10nM testosterone 24 h after transfection period. Values are mean ±S.D. from three independent experiments. * p< 0.01, ** p<0.001, two-way anova test.C) Cell proliferation in PC3 cells transfected with GPR56-siRNA. Cell proliferation in PC3 cells and cells transfected with siGPR56 were examined at 24 h, 48 h, 72 h or 96 h after seeding, using MTT assay performed in charcoal stripped medium. The data represents the mean ±S.D. of three independent experiments. Values are mean ±S.D. from three independent experiments. * p< 0.01, ** p<0.001, *** p<0.0001, two-way anova test. (TIF) [file pone.0226056.s003.tif]

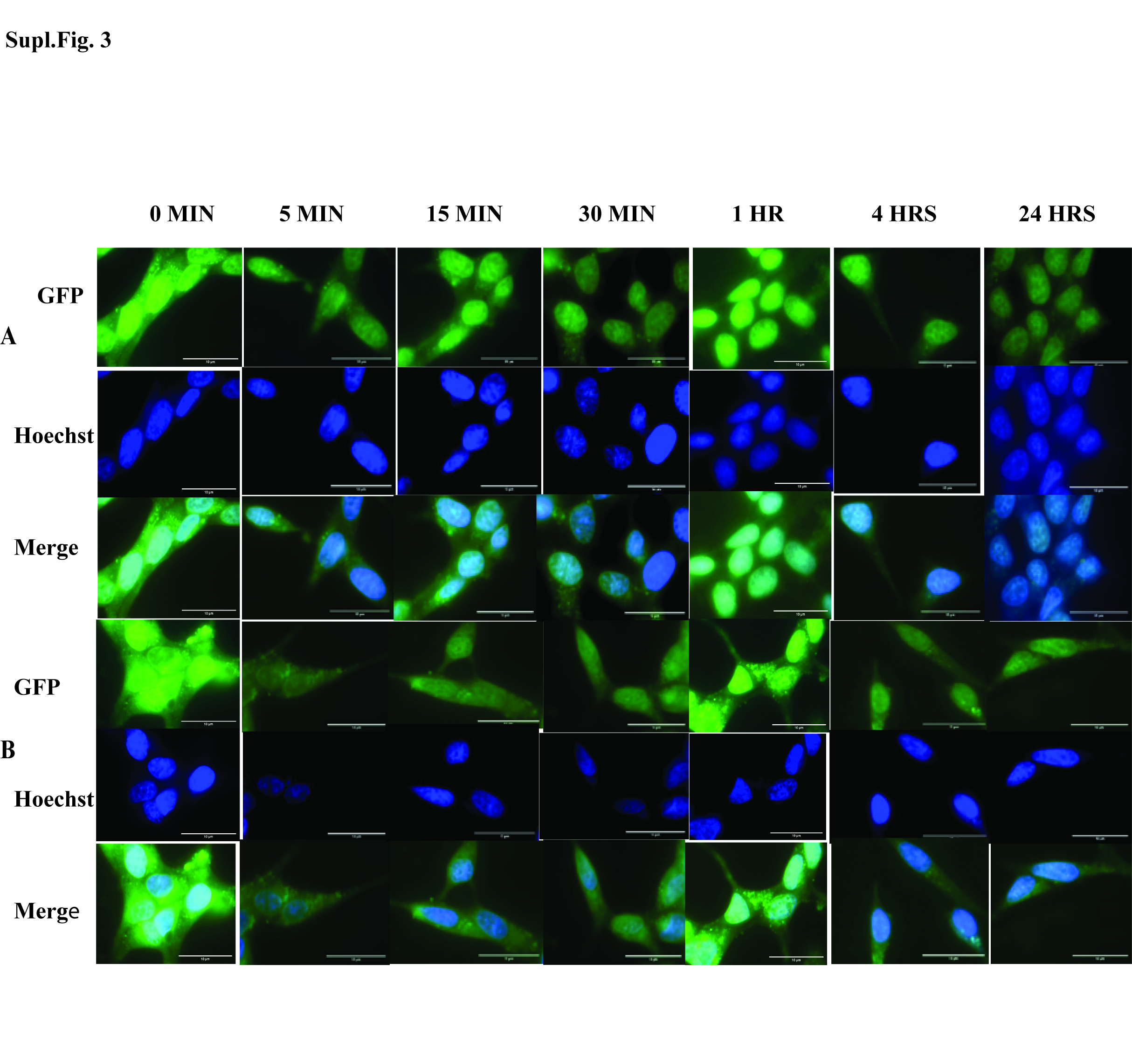

Supplement: S3 Fig — A-B) Knockdown of GPR56 expression causes inhibition of AR translocation: LNCaP cells were transfected using GFP-AR, or GFP-AR along with siGPR56 (100nM), and treated with 10 nM T 24 h after transfection. Fluorescence Images captured at various time intervals 5 mins, 15 mins, 30 mins, 1 hour, 4 hours, 24 hours. Nuclei were visualized by Hoechst staining. Scale bar,10um (added using Image J). (TIF) [file pone.0226056.s004.tif]
