# Supplementary material for: Activation of GPR56, a novel adhesion GPCR, is necessary for nuclear androgen receptor signaling in prostate cells
Source: PLoS One. 2020 Sep 3;15(9):e0226056. doi: 10.1371/journal.pone.0226056 (PMC7470385; doi:10.1371/journal.pone.0226056)

Fig 2A. Beta actin (60 bp) in cell lines. The top bands in the gel is beta actin (Lane 2- lane 6) pls ignore the lower band as it is different gene PCR but run on the same gel.

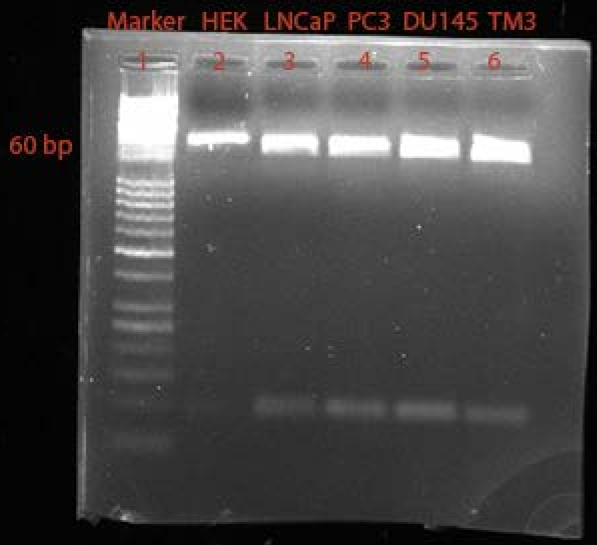

Fig 2A: GPR56 mRNA expression in cell lines (100 bp). The highlighted band is GPR56 (Lane 3-Lane 6)

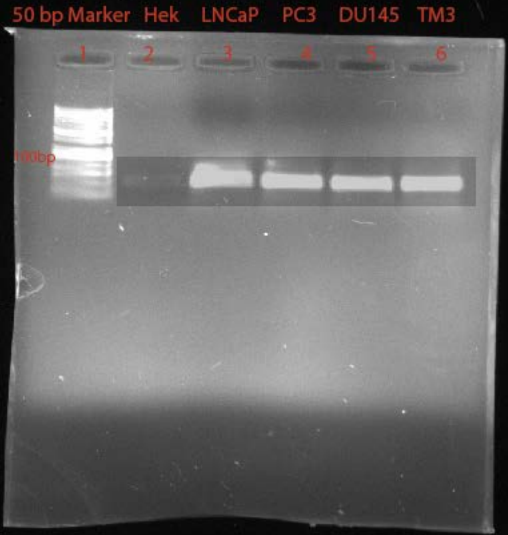

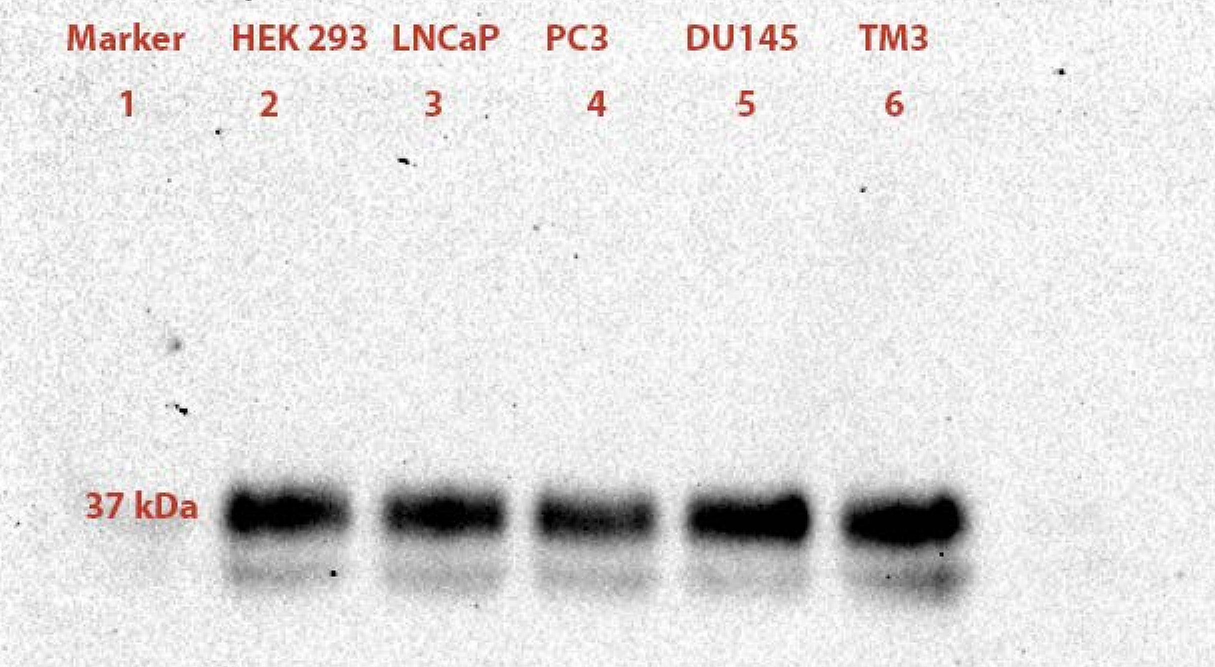

Fig 2 B. GAPDH (37KDa) (positive control) protein expression in cell lines

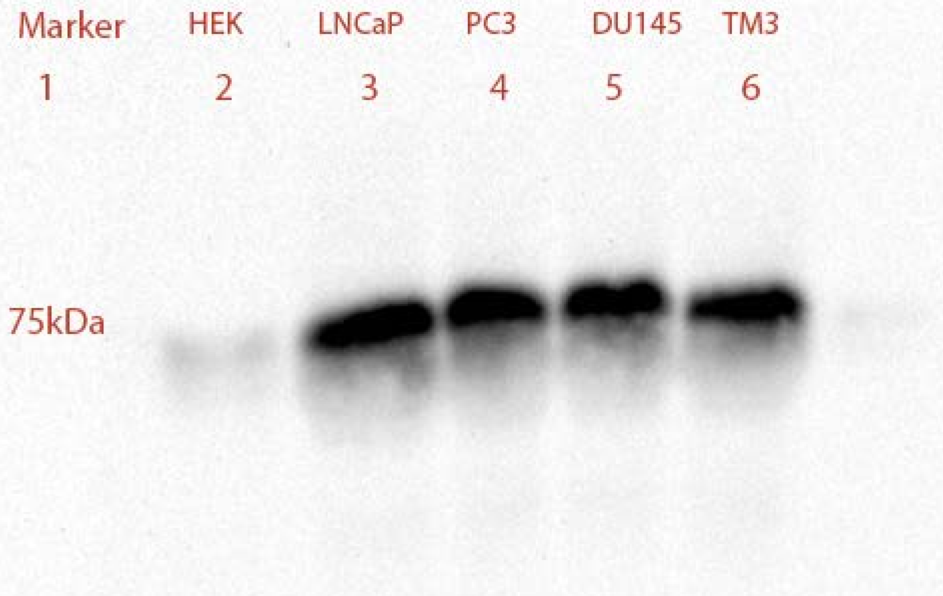

Fig 2 B GPR56 (75 KDa) protein expression (Lanes 3-6) seen in cell lines.

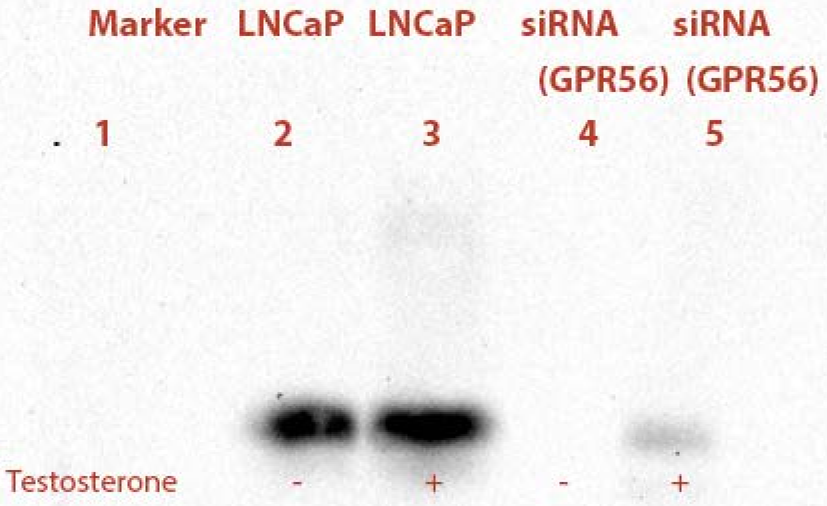

Fig 2C. GPR56 (75 KDa) protein expression (75 KDa) in LNCaP cells when siRNA against GPR56 was used

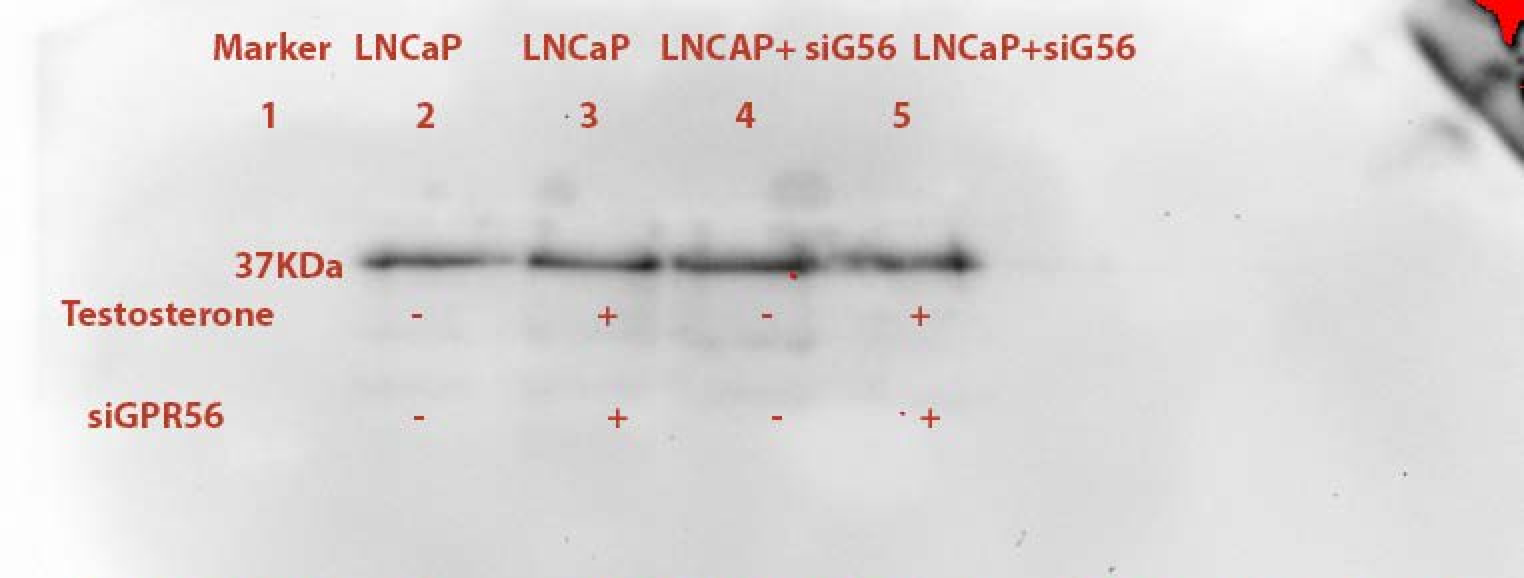

Fig 2C. GAPDH (positive control) (37 KDa) in LNCaP cells and LNCaP cells transfected with siRNA against GPR56 was used

Fig 3J Beta actin (60 bp) mRNA in LNCaP cells, LNCaP cells transfected with siRNA against GPR56, scrambled siRNA

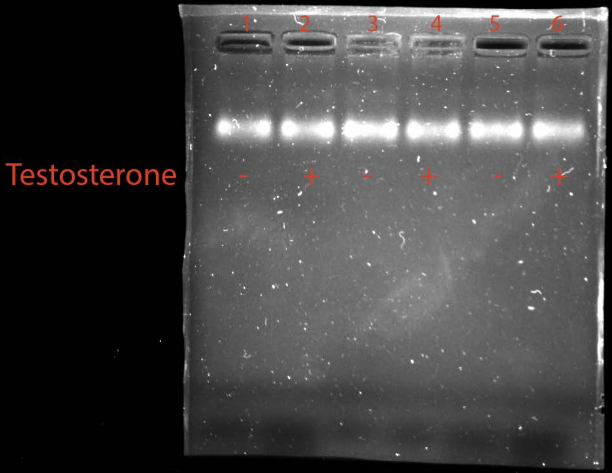

Fig 3J. TMPRSS2 (110bp) mRNA expression in LNCaP cells, LNCaP transfected siRNA against GPR56 or scrambled siRNA. The top bands are selected as per the size and lower bands are primer dimer.

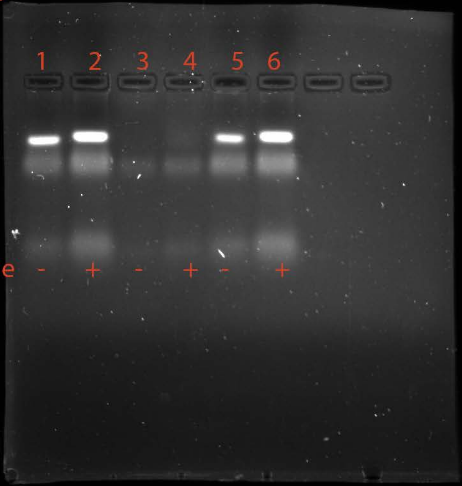

Fig 3J: PSA (250 bp) expression in LNCaP cells, LNCaP cells transfected with siRNA against GPR56 or Scrambled siRNA against GPR56. The lower band is selected as each time on PCR the above band is seen and lower band gives the desired size.

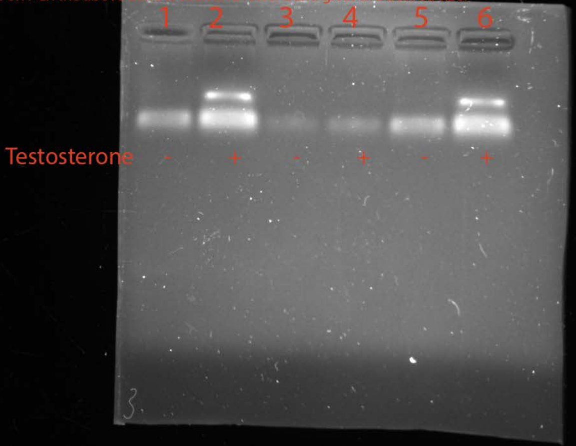

Fig 3J: GPR56 (100bp) expression in LNCaP cells, LNCaP cells transfected with siRNA against GPR56 or Scrambled siRNA. The above band is selected as per the size and lower band is primer dimer.

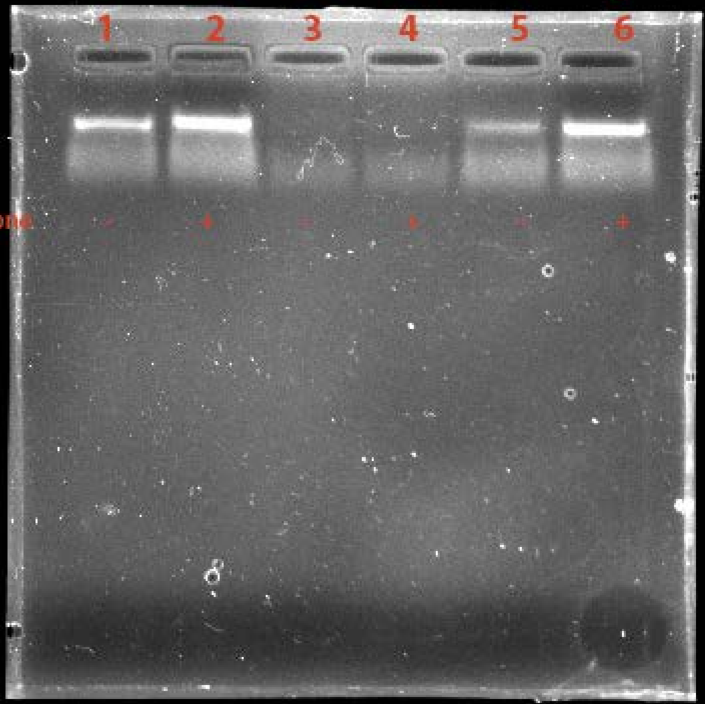

Fig 5B (Total lysate of Hek cells, HEK cells transfected with GPR56, GPR56 (NT), GPR56 (NT), GPR56 (CT), GPR56 (SD2)

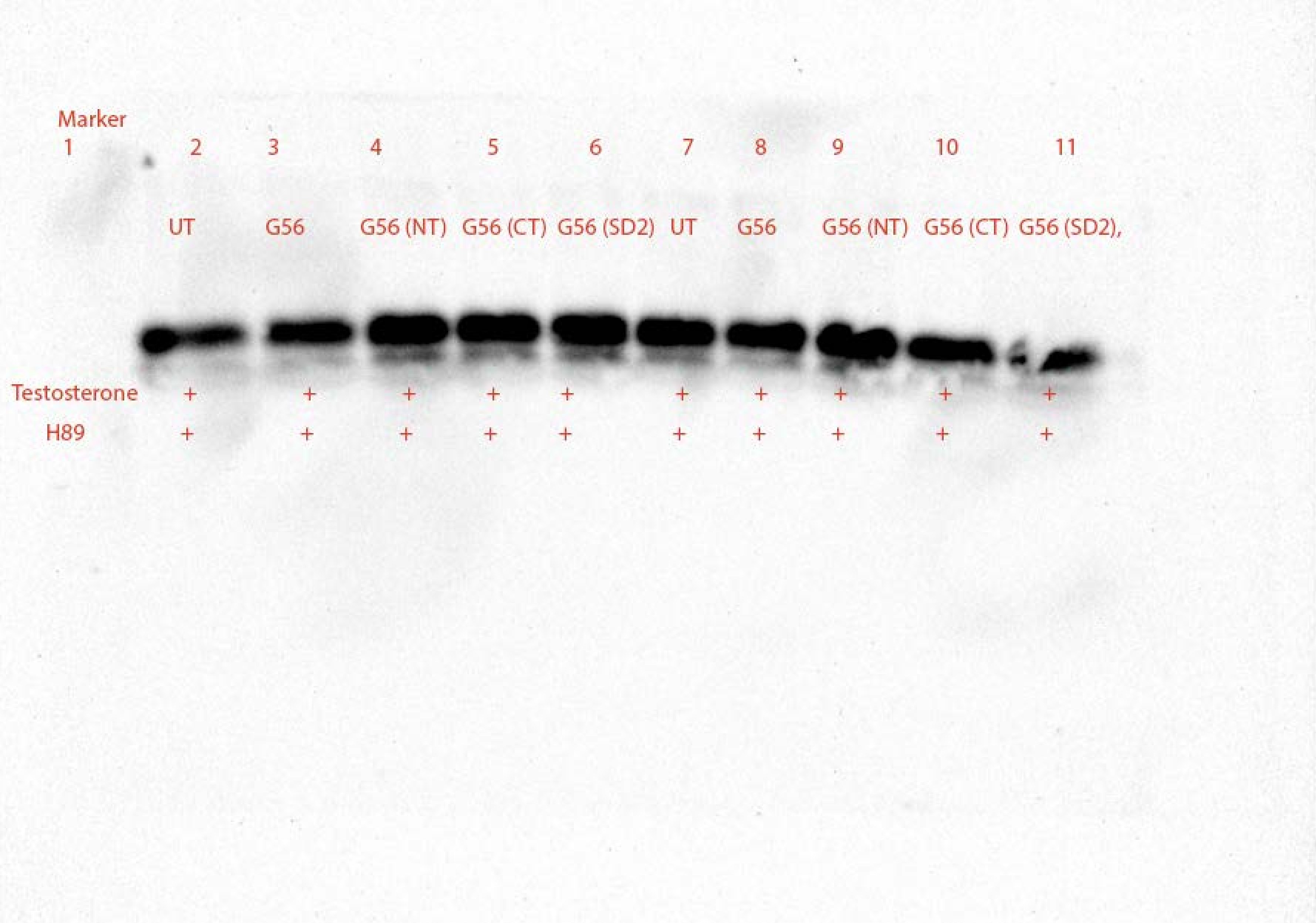

Fig 5A. GTP Rho activation in GPR56 transfected HEK293 cells, Ga13siRNA or scrambled siRNA.

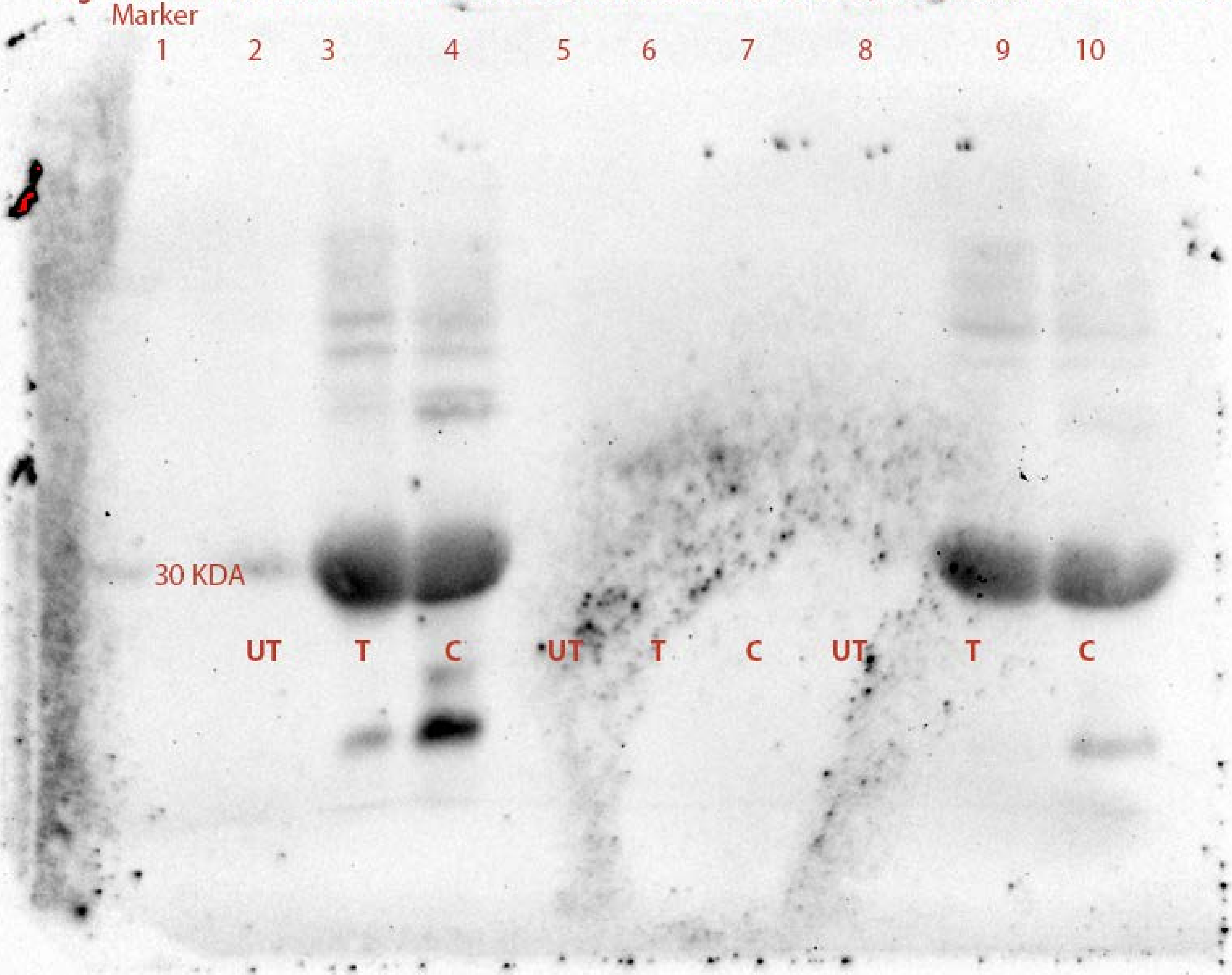

Fig 5A. Total lysate for GTP Rho assay in HEK cells transfected with GPR56, Ga siRNA or scrambled siRNA

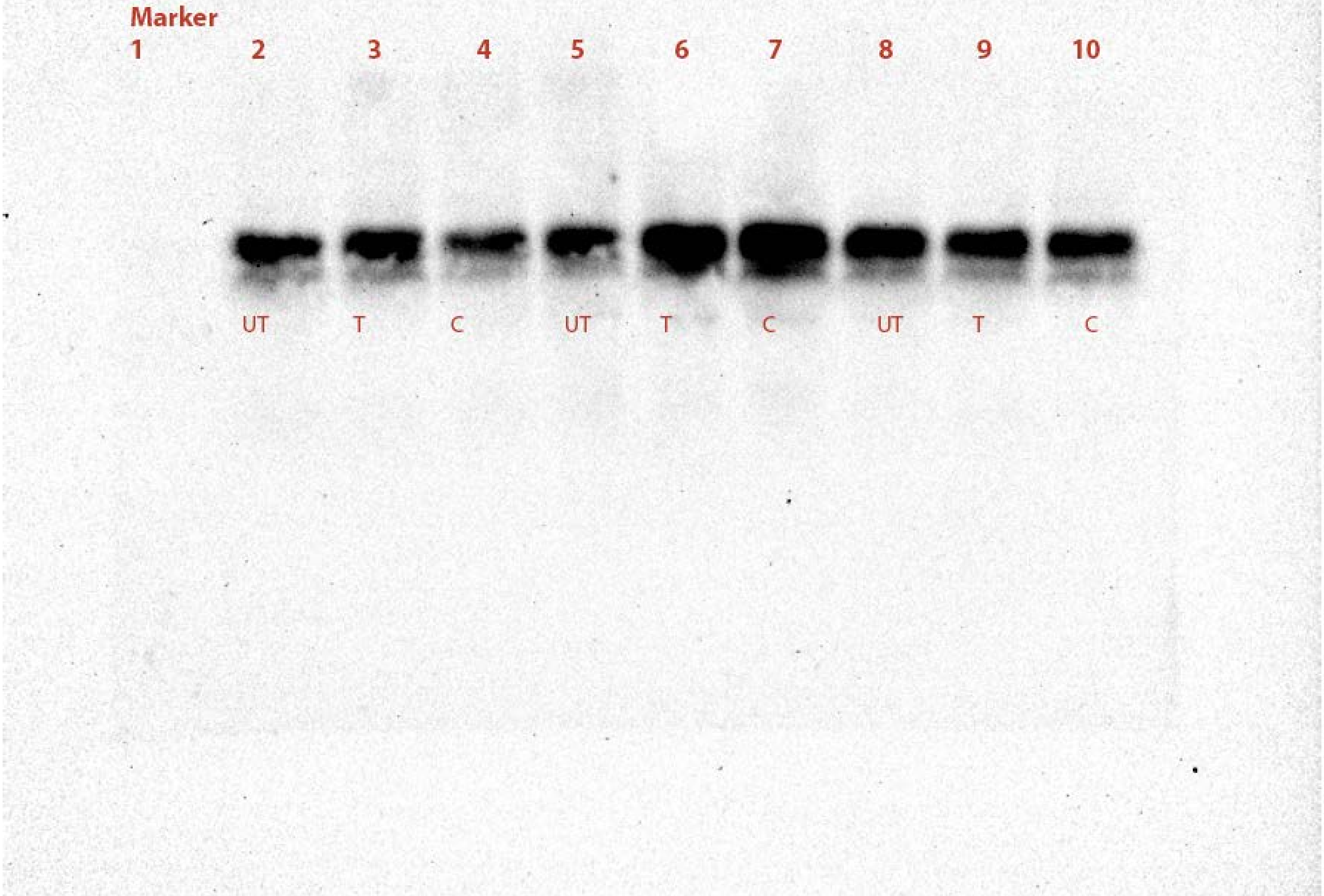

Fig 5B GTP Rho activation in HEK cells and Hek cells transfected with GPR56 and its mutants (GPR56(NT), GPR56 (CT), GPR56 (SD2)

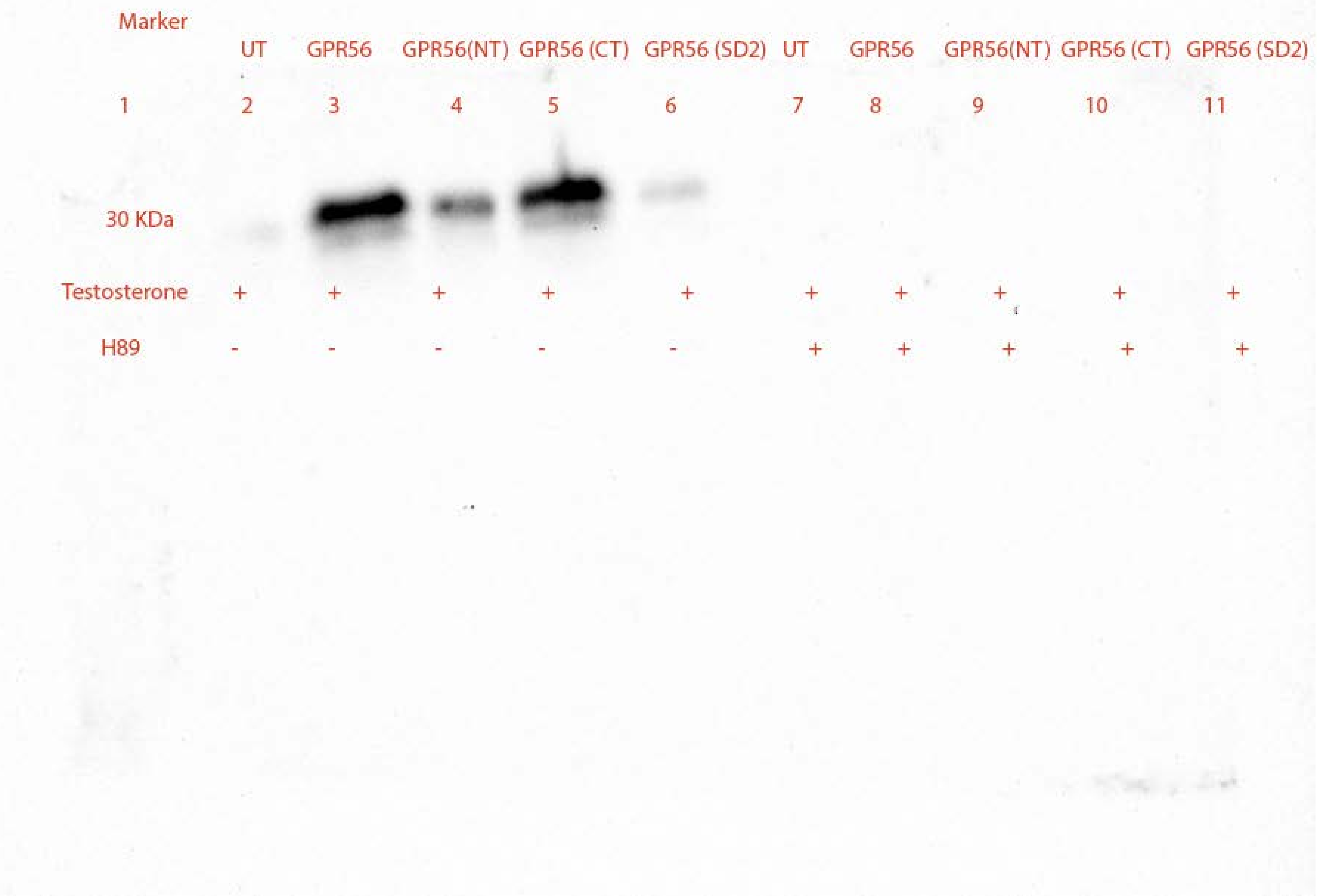

Fig 6B. AR translocation in LNCaP cells, LNCaP cells tranfected wit
siRNA against GPR56

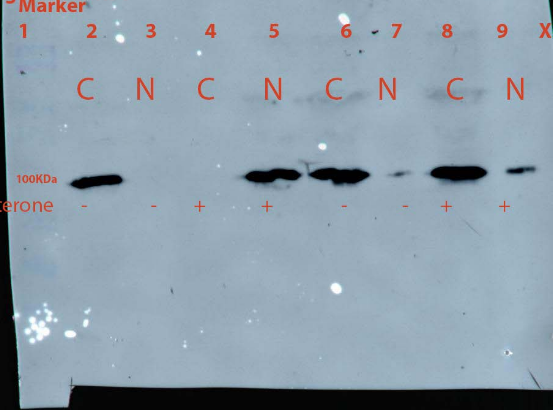

Fig 6B. GAPDH as positive control (AR translocation) in LNCaP cells, LNCaP cells transfected with siRNA against GPR56.

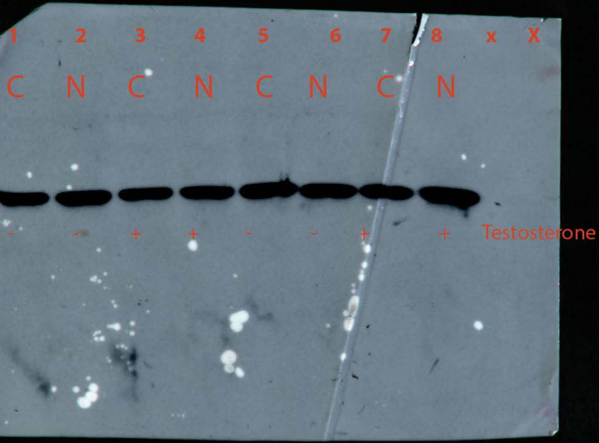

Fig 7 D. Beta actin (60 bp) in patients' tissue samples (P1-P10). Ladder is not run as limited wells and size is known for beta actin (i.e.60 bp). The top band is selected.

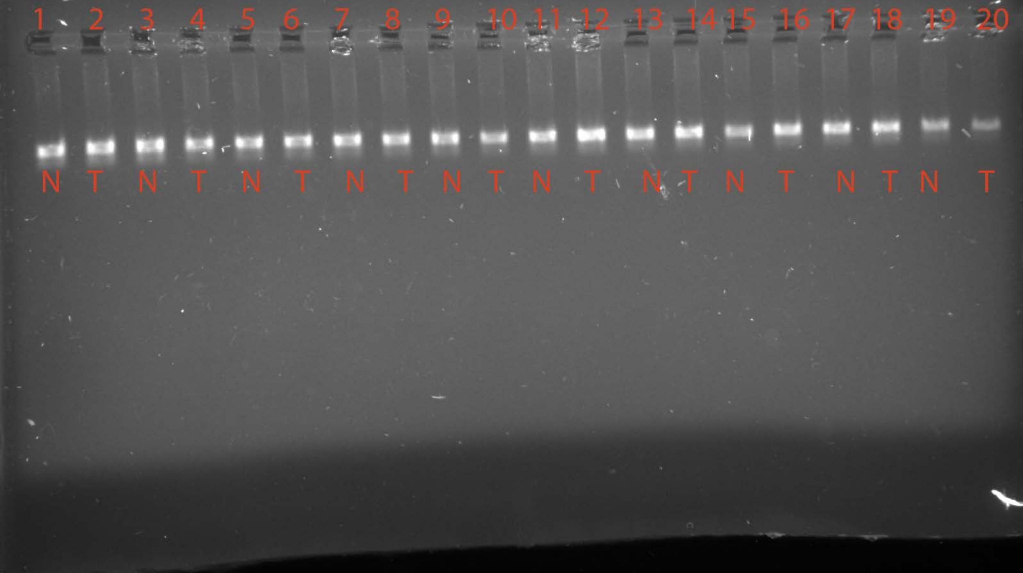

Fig 7D. GPR56 (NT) (113 bp) mRNA expression in patients' tissue samples (P1-P10)

The prominent band is selected based on the size which is 113 bp.

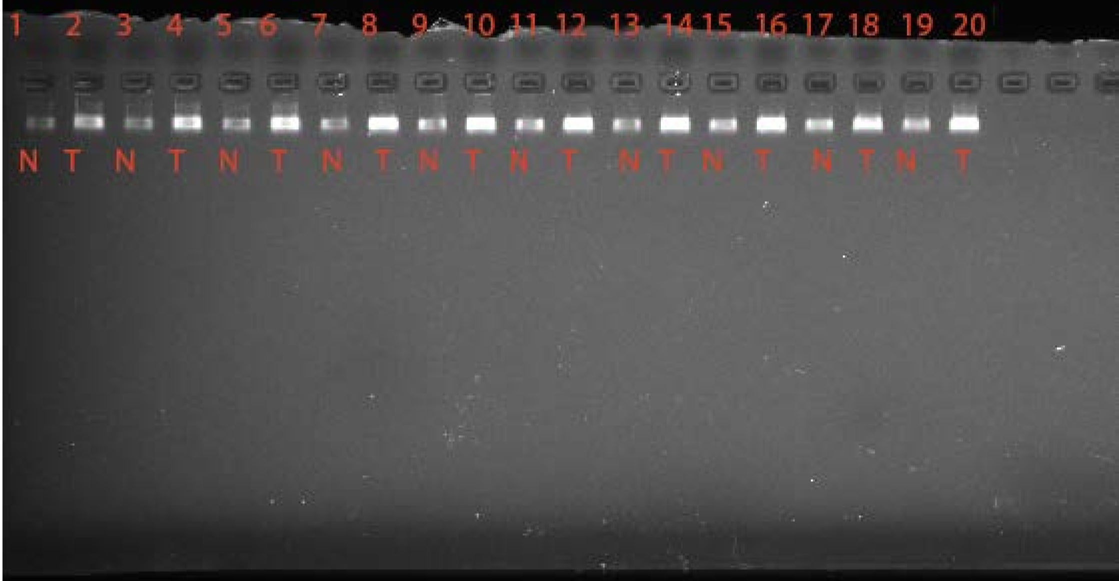

Fig 7D. GPR56 (CT) (100bp) mRNA in patients' tissue samples (P1-P10). The highlighted band is selected as below bands are primer dimers and the size of GPR56 (CT) should be (100bp).

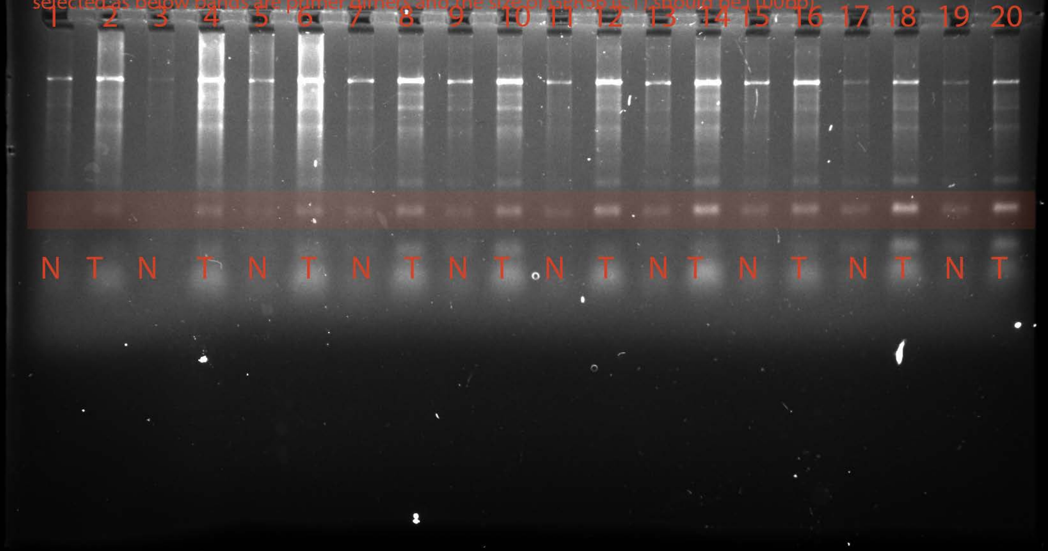

Fig 8 A. GPR56 protein expression in patients' samples (P1-P5). Ladder is not run as size expected is 75 KDa

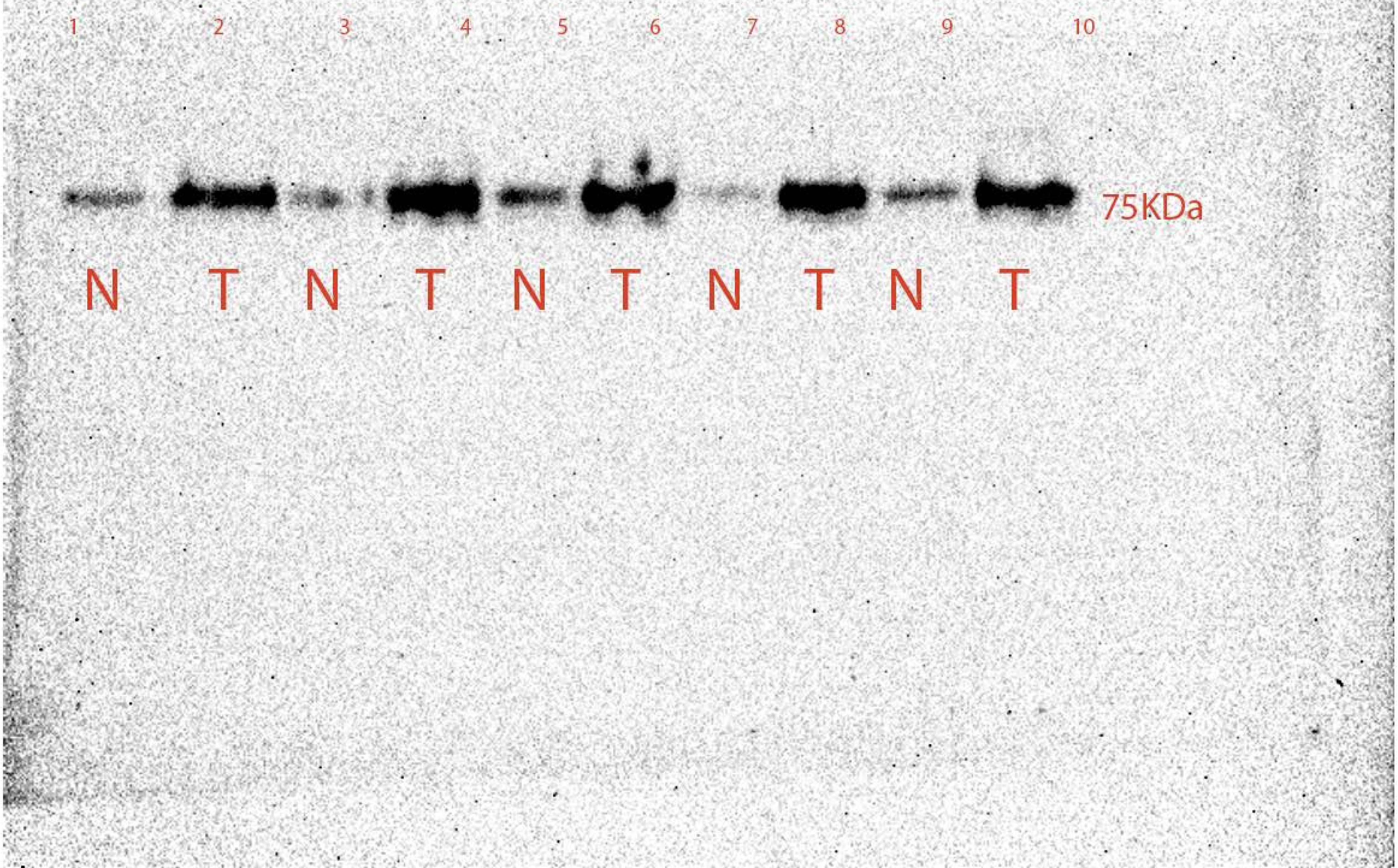

Fig 8 A. GAPDH (37kDa) (positive control) protein expression in patients' samples (P1-P5)

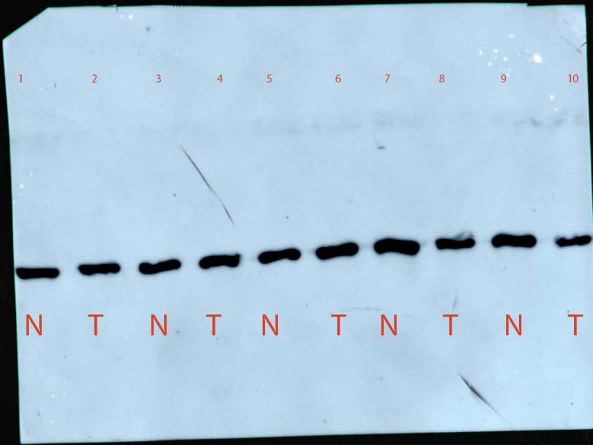

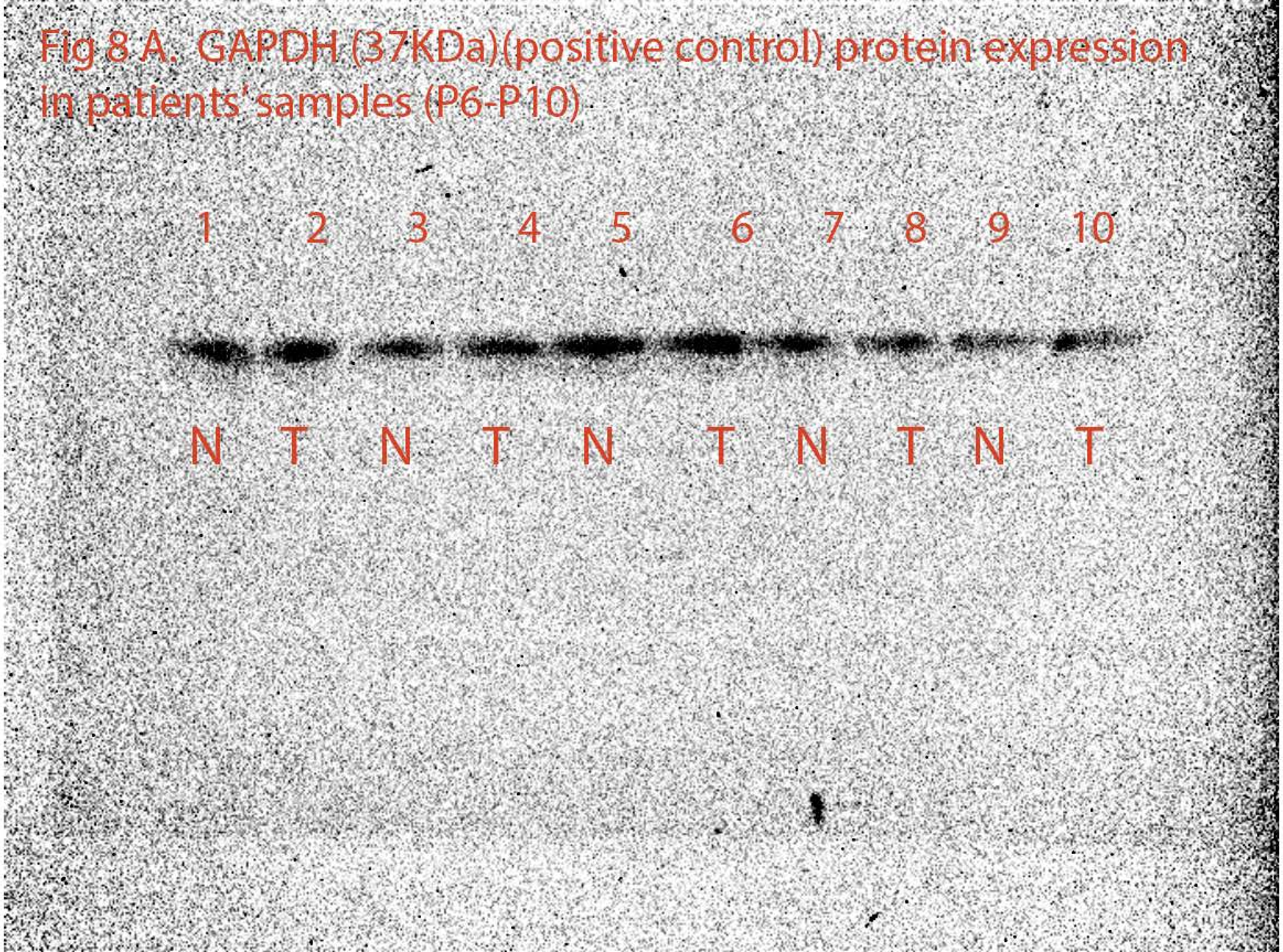

Fig 8 A. GAPDH (37KDa)(positive control) protein expression in patients' samples (P6-P10)

Fig 8 A. GPR56 protein expression in patients' samples (P6-P10) . Ladder is not run as size expected is 75 KDa

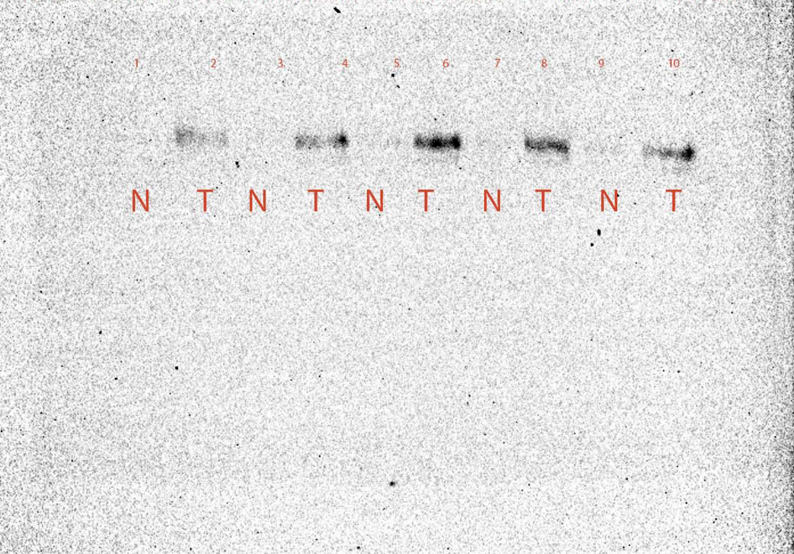

Supplement: S1 Raw images — (PDF) [file pone.0226056.s005.pdf]
