# Supplementary material for: Activation of GPR56, a novel adhesion GPCR, is necessary for nuclear androgen receptor signaling in prostate cells
Source: PLoS One. 2020 Sep 3;15(9):e0226056. doi: 10.1371/journal.pone.0226056 (PMC7470385; doi:10.1371/journal.pone.0226056)
Supplement: S2 Raw images — (PDF) [file pone.0226056.s006.pdf]

Fig 6c Cytoplasmic and nuclear fractionation of AR in unstimulated (vehicle treated) and testosterone stimulated LNCaP cells in presence and absence of GPR56 siRNA (siGPR56).

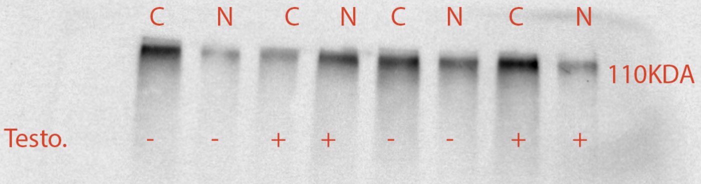

Fig 6c GAPDH as cytoplasmic marker in LNCaP cells and LNCaP cells transfected with siRNA against GPR56

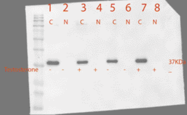

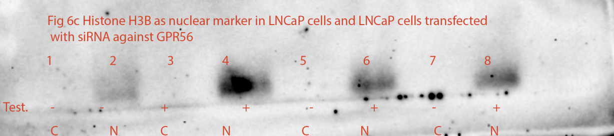

Fig 6c Histone H3B as nuclear marker in LNCaP cells and LNCaP cells transfected with siRNA against GPR56
