# Supplementary material for: Activation of GPR56, a novel adhesion GPCR, is necessary for nuclear androgen receptor signaling in prostate cells
Source: PLoS One. 2020 Sep 3;15(9):e0226056. doi: 10.1371/journal.pone.0226056 (PMC7470385; doi:10.1371/journal.pone.0226056)
Supplement: Attachment — Submitted filename: Plos one - Response to reviewers comments (1).docx [file pone.0226056.s007.docx]

Dear Editor,

Thank you for considering our article. Also, thanks to the reviewers who have read the manuscript meticulously and provided constructive comments. According to the reviewers’ suggestions, we have now performed more experiments and have included them in the manuscript text and figures. The pointwise response is also included below.

**Reviewer #1:**

Singh and coworkers conducted a study entitled “Activation of GPR56, a novel adhesion GPCR, is necessary for nuclear androgen receptor signaling in prostate cells” in which the authors indicated that androgens activate Rho-signaling pathway through the activation of GPR56. GPR56 knockdown blocks the nuclear translocation of AR and its downstream transcription factors. To validate the in vitro studies, GPR56 was upregulated in prostate cancer (PCa) tumor specimens when compared with normal tissues.

General comments:
The manuscript was well-designed, technical aspects used in this study are appropriate, results are supported with enough data and the size of the text is reasonable. However, I have some few comments need be addressed in the current study.
1. - Using LNCaP as a model is appropriate here but PC-3 representing CRPC cells should be also included as a control.

Response: The PC3 cell line has now been used along with LNCaP cells for performing Luciferase assay and MTT assay and the results have now been included in the text and figures ( 3H and 7C). It is important to note that the response in PC3 cells is similar to that of LNCaP.

2. Examining the expression of GPR56 transcript in prostate cancer tissue is not enough because posttranslational modification can change the whole story. It is better to stain the tissue with GPR56 specific antibody.

Response: To demonstrate the changes in GPR56 expression at the protein level in normal versus tumour tissues, Western Blot analysis has been performed using normal and tumour tissues using GPR56 antibody. The result is now included as Figure 8A and the intensity quantitation is also depicted. The expression of GPR56 is significantly higher at the protein level in all tumour tissues analysed.

3.In addition, the authors showed that GPR56 triggers cytosolic translocation of AR using immunofluorescence, and it would be more believable if the authors use cell fractionation to validate the results.

Response: As per the reviewer’s suggestion, we have now performed Western Blot experiment using nuclear and cytoplasmic fractions of LNCaP cells transfected using scrambled siRNA or GPR56 siRNA, and probed using anti-AR antibody and the results have been included as Fig. 6C. The results clearly demonstrate that in LNCaP cells expressing GPR56, the AR is in the cytoplasm in untreated cells, but moves to the nucleus on testosterone treatment, whereas in the cells transfected using GPR56 siRNA, upon testosterone stimulation, a significant proportion of AR continues to reside in the cytoplasm and does not migrate into the nucleus.

Specific comments:
- In the abstract: Line 4, the phrase of “a role for these in CRPC” should be fixed.

Response: This sentence has now been corrected.

Please include bioinformatics results of AR-ligand similarity and the number of examined PCa tissues (25 tissue samples) in the abstract.

Response: The details have now been included in the Abstract.

- In Methods section, please replace (u: micron) with Latin symbol (µ).

Response: Corrected.

Clinical characteristics of human prostate cancer samples are needed.

Response: Clinical Characteristics of patients have now been included in the M and M section as Supplementary Table 1.

- In results section, all figures need an improvement because of quality was very poor.

Response: High resolution images for all figures have now been included in Tiff format 300 Dpi.

Figure legends must be included together after the MS text not included in the results.

Response: Figure legends have now been included after the MS text.

In results, page 14, please replace S 1 Fig. with Supl. Fig. 1.

Response: Corrected.

Statistical analysis should be included in Figure 7A&B.

In Fig. 7C, quantification of band densities will improve the results of GRP56 in tissues samples.

Response: Statistical Analysis has now been included for Fig. 7A, B and C and quantification of band densities has also been included for Fig. 7D.
- In discussion section, page 21, “Cancerous prostate” should be replaced.

Response: Has been replaced.

I suggest to move Figure 8 A&C and also Tables 3&4 to the results section (The Authors should discuss the results but not to include new results).

Response: Figures 8A and C have now been moved to Results section as Fig. 3A and Fig. 8B.

Tables 3 and 4: Have now been moved to Results Section

At the end of discussion, summary or conclusion should be include in which you summarize your findings.

Response: A Summary section has now been included.

Discussion should be more concise, focus and comprehensive.
- The manuscript should be edited again because it has several typos and incorrect structures.
Response: We have edited the MS again to correct typos.

**Reviewer #2:**

In the current manuscript titled “Activation of GPR56, a novel adhesion GPCR, is necessary for nuclear androgen receptor signaling in prostate cells” demonstrates the role of GPR56 in regulating AR downstream signaling. The authors have demonstrated how binding of the testosterone to the GPR56 can activate PKA and Rho signaling promoting AR nuclear translocation demonstrating crosstalk between two signaling pathways. However, following concerns need to be addressed prior to publication.
1- Since CRPC occurs in patients having very low levels of testosterone it does not explain how GPR56 can lead to CRPC which the authors are claiming (the docking score for AR is much higher for testosterone than GPR56, table 2) this needs to be clarified in the discussion.

Response: While in CRPC testosterone levels are reduced, yet the expression of most GPCRs and their ligands are significantly enhanced (Daaka, 2005). Increase in expression of GPR56 in tumour samples over their normal counterparts has been demonstrated in this study (Fig.7D and 8A) and through TCGA analysis of previous studies (Fig. 8B). Increased expression of GPR56 via any means is likely to result in increased activation of PKA, which would result in increased nuclear migration of AR due to phosphorylation of HSP90 (Dagar et al, 2019). This would result in enhanced cell proliferation in low/no testosterone conditions as observed in CRPC. This has now been explained more clearly in Discussion section.

2- AR receptor signaling studies require the testosterone induction to be performed in phenol red free charcoal stripped serum media, authors have used serum starvation instead that is not ideal for these studies.

Response: In literature, some studies have used serum starvation while others have used charcoal stripped serum. We repeated few of the experiments in Charcoal stripped serum to verify that the results were similar to those obtained with serum starvation. These results are now included as Supplementary Figure 2.

3- The authors need to demonstrate if GPR56 siRNA can block AR nuclear localization using cell fractionation studies.

Response: This has now been included in the text and Results section as Figure 6C.

4- Fig3H-The PSA levels go down after testosterone treatment whereas GPR56 expression go up. Testosterone upregulates AR signaling and PSA expression, authors need to explain this abnormal result. Also they need to show effect on another AR regulated gene.

Response: To verify the effect of GPR56 knockdown, LNCaP cells were transfected with GPR56 siRNA or scrambled and expression levels of genes such as PSA and GPR56 was analysed.

This experiment has now been repeated to verify the results and expression of both PSA and GPR56 are upregulated upon Testosterone treatment. Expression of another androgen target gene, namely TMPRSS2 was also analysed and found to increase upon Testosterone treatment. Expression of all the three genes were negligible in LNCaP cells transfected using GPR56 siRNA.

This has now been included in text, page number 16.

Minor concerns:
1- Authors have included the figure legends in between the manuscript text without the figure that is not reader friendly.

Response: Figure legends have now been included at the end of the text.
2- Constructs- is it at the HindIII site or between HindIII and another restriction enzyme?

Response: It is at the Hind III site and has been corrected in the M and M section.
3- The GTP Rho pull down experiment not described properly in methods section.

Response: Has now been explained in detail.
4- Was the cyclic AMP assay performed 2-4 hrs after serum starvation or after 24 hrs of starvation?

Response: This has now been corrected in the M and M section regarding cAMP assay.
5- Page 15, correct that GPR56 protein is not expressed in HEK cells

Response: This has now been corrected and included on page 15.
6- Fig 3F-Is the control scrambled siRNA?

Response: The results were similar in both LNCaP cells wildtype and that transfected with scrambled GPR56 siRNA. In the figure results for LNCaP cells transfected using scrambled GPR56 has been included.
7- Rephrase “androgen transcription” to AR downstream signaling, authors are not looking into AR mRNA levels.

Response: Has now been rephrased.
8- Fig4A-PKAsiRNA set is still showing response to ISO.

Response: The experiment with Isoproterenol has been repeated and the results of PKA activity have been revised.
9- Fig 7- provide a western blotting with N and C terminal GPR56 antibodies.

Response: The Western Blot experiment has now been performed using GPR56 N-terminal antibody (C-terminal antibody was not available at short notice) and the results have been modified accordingly in text and included as Fig. 8A.

We would appreciate receiving your revised manuscript by Feb 28 2020 11:59PM. When you are ready to submit your revision, log on to https://www.editorialmanager.com/pone/ and select the 'Submissions Needing Revision' folder to locate your manuscript file. If you would like to make changes to your financial disclosure, please include your updated statement in your cover letter.

To enhance the reproducibility of your results, we recommend that if applicable you deposit your laboratory protocols in protocols.io, where a protocol can be assigned its own identifier (DOI) such that it can be cited independently in the future. For instructions see: <http://journals.plos.org/plosone/s/submission-guidelines#loc-laboratory-protocols>

Please include the following items when submitting your revised manuscript:

- A rebuttal letter that responds to each point raised by the academic editor and reviewer(s). This letter should be uploaded as separate file and labeled 'Response to Reviewers'.
- A marked-up copy of your manuscript that highlights changes made to the original version. This file should be uploaded as separate file and labeled 'Revised Manuscript with Track Changes'.
- An unmarked version of your revised paper without tracked changes. This file should be uploaded as separate file and labeled 'Manuscript'.

Please note while forming your response, if your article is accepted, you may have the opportunity to make the peer review history publicly available. The record will include editor decision letters (with reviews) and your responses to reviewer comments. If eligible, we will contact you to opt in or out.

We look forward to receiving your revised manuscript.

Kind regards,

Mohammad Saleem

University of Minnesota
Academic Editor, PLOS ONE

Journal Requirements:

When submitting your revision, we need you to address these additional requirements.

1. Please ensure that your manuscript meets PLOS ONE's style requirements, including those for file naming. The PLOS ONE style templates can be found at http://www.journals.plos.org/plosone/s/file?id=wjVg/PLOSOne_formatting_sample_main_body.pdf and http://www.journals.plos.org/plosone/s/file?id=ba62/PLOSOne_formatting_sample_title_authors_affiliations.pdf

2. PLOS ONE now requires that authors provide the original uncropped and unadjusted images underlying all blot or gel results reported in a submission’s figures or Supporting Information files. This policy and the journal’s other requirements for blot/gel reporting and figure preparation are described in detail at https://journals.plos.org/plosone/s/figures#loc-blot-and-gel-reporting-requirements and https://journals.plos.org/plosone/s/figures#loc-preparing-figures-from-image-files. When you submit your revised manuscript, please ensure that your figures adhere fully to these guidelines and provide the original underlying images for all blot or gel data reported in your submission. See the following link for instructions on providing the original image data: https://journals.plos.org/plosone/s/figures#loc-original-images-for-blots-and-gels.

In your cover letter, please note whether your blot/gel image data are in Supporting Information or posted at a public data repository, provide the repository URL if relevant, and provide specific details as to which raw blot/gel images, if any, are not available. Email us at plosone@plos.org if you have any questions.

3. PLOS requires an ORCID iD for the corresponding author in Editorial Manager on papers submitted after December 6th, 2016. Please ensure that you have an ORCID iD and that it is validated in Editorial Manager. To do this, go to ‘Update my Information’ (in the upper left-hand corner of the main menu), and click on the Fetch/Validate link next to the ORCID field. This will take you to the ORCID site and allow you to create a new iD or authenticate a pre-existing iD in Editorial Manager. Please see the following video for instructions on linking an ORCID iD to your Editorial Manager account: https://www.youtube.com/watch?v=_xcclfuvtxQ

4. We note that you have included the phrase “data not shown” in your manuscript. Unfortunately, this does not meet our data sharing requirements. PLOS does not permit references to inaccessible data. We require that authors provide all relevant data within the paper, Supporting Information files, or in an acceptable, public repository. Please add a citation to support this phrase or upload the data that corresponds with these findings to a stable repository (such as Figshare or Dryad) and provide and URLs, DOIs, or accession numbers that may be used to access these data. Or, if the data are not a core part of the research being presented in your study, we ask that you remove the phrase that refers to these data.
