# Supplementary material for: Activation of GPR56, a novel adhesion GPCR, is necessary for nuclear androgen receptor signaling in prostate cells
Source: PLoS One. 2020 Sep 3;15(9):e0226056. doi: 10.1371/journal.pone.0226056 (PMC7470385; doi:10.1371/journal.pone.0226056)
Supplement: Attachment — Submitted filename: Rebuttal Letter 2nd July.docx [file pone.0226056.s008.docx]

Dear Editor,

We thank you for the review of the article. We are happy to see that the reviewers were satisfied on almost all accounts.

As per the request of Reviewer 2, point 6, a fresh Western blot experiment has been performed, where presence of androgen receptor (AR) in the cytoplasmic and nuclear fraction was probed. As control we have probed the blot using both GAPDH (cytoplasmic marker) and Histone H3B (nuclear marker). While GAPDH expression is observed in cytoplasmic fractions only, Histone H3B expression was restricted to the nucleus.

Accordingly, changes have been made in the text and the figure has been replaced. In addition, the name of Mr. Sudhir Kumar has been added to the list of authors as he helped us in performing the last experiment. Also, the Histone H3B antibody was a kind gift from Dr. Rakesh Saxena whose name has now been added in acknowledgement and Materials and Methods.

Hope the manuscript is acceptable to you in its current form.

Thanks, and regards,

Gargi Bagchi
